# Posterior Reversible Leukoencephalopathy Syndrome in a Patient after Acute COVID-19 Infection

**DOI:** 10.1155/2021/5564802

**Published:** 2021-04-23

**Authors:** Ketino Kobaidze, Yoo Mee Shin, Mariam Japaridze, Ioannis Karakis, Xin Wu

**Affiliations:** ^1^Associate Professor of Medicine, Emory University School of Medicine, Division of Hospital Medicine 550 Peachtree Street, Atlanta, GA 30308, USA; ^2^Division of Hospital Medicine, Emory University School of Medicine, Atlanta, GA 30308, USA; ^3^Ross University School of Medicine, New Jersey, USA; ^4^Department of Neurology, Emory University School of Medicine, Atlanta, GA 30308, USA; ^5^Department of Radiology and Imaging Science, Emory University School of Medicine, Atlanta, GA 30308, USA

## Abstract

The SARS-CoV-2 infection affects numerous organs, including the central nervous system. The neuroinvasive abilities and neuroinflammation may lead to short- and long-term neurological manifestations. Among neurological disorders associated with SARS-CoV-2 infection, posterior reversible encephalopathy syndrome (PRES) has been described in a few case-based observational studies during the acute phase of COVID-19 hospitalization. We present a case of a patient who developed seizures and PRES after recovering from an acute severe COVID-19 infection.

A 90-year-old African American female with multiple comorbidities and a severe COVID-19 infection was discharged home in stable condition after two weeks of hospitalization. A week later, she developed new-onset generalized tonic-clonic seizures requiring readmission to the hospital. The patient's clinical course and brain imaging supported PRES. Her mentation returned to baseline with supportive care and anticonvulsant treatment. Follow-up brain MRI four months later demonstrated resolution of FLAIR signal abnormalities confirming PRES. SARS-CoV-2 insult on the cerebrovascular endothelial cells likely continued and despite the clinical recovery eventually resulted in PRES. We believe that this is the first case describing the presentation of PRES after recovery from severe acute COVID-19 infection.

## 1. Introduction

Posterior reversible encephalopathy (PRES) is a neurological syndrome of cerebral dysautoregulation which can occur in multiple settings, including but not limited to acute migraine, occipital epilepsy, eclampsia/preeclampsia, hypertensive encephalopathy, and the use of cytotoxic and immunosuppressive medications [1]. While helpful in establishing the diagnosis, neuroimaging studies are unable to distinguish between the varying etiologies. Disruption of cerebral autoregulation combined with endothelial dysfunction appears to play important roles in the development of this clinical condition, which has been seen to be a result of transitional vasospasm, ischemic injury, or vasogenic edema, due to increased capillary permeability. Patients in all age groups are susceptible, though it has a slightly higher predilection for women.

The currently ongoing novel COVID-19 pandemic has affected millions of individuals across the globe, and there are over 539, 038 fatal cases reported in the United States alone [2]. Widespread dysregulation of pulmonary, renal, cardiac, and circulatory homeostasis contributes to morbidity and mortality in COVID-19 patients [3]. Knowledge about COVID-19's clinical presentation and disease progression is rapidly growing. However, less known are longer-term outcomes, especially for patients who are discharged from hospitals after severe acute COVID-19 infection.

There is already ample evidence that neurological complications of SARS-CoV-2 are common and may precede respiratory symptoms [4–8]. The expression of angiotensin-converting enzyme 2 (ACE-2) in brain vasculature creates an opportunity for the virus to enter into CNS cells, resulting in harm to neurological tissue [6, 9, 10]. It alters the blood-brain barrier permeability, which may result in brain autoregulation and vascular circulation disruption [11]. Several neurological complications of COVID-19 infection have been described in the literature. Patients may present with headache, acute encephalopathy, encephalitis, ischemic or hemorrhagic stroke, demyelinating neuropathy (Guillain–Barre syndrome), anosmia, ageusia, and seizures [3, 6, 7, 9].

Endothelial dysfunction in the setting of COVID-19 may contribute to PRES, as this complication has been described among hospitalized patients with COVID-19 [5]. Neurologic effects of SARS-CoV-2 may have long-term consequences. We present a case of an elderly patient who developed seizures and PRES after recovering from the acute phase of COVID-19 infection. We believe that this rare complication was associated with the recent SAR-CoV-2 virus infection affecting the nervous system.

## 2. Case Presentation

A 90-year-old African American female with a history of type 2 diabetes, essential hypertension, deep venous thrombosis, pulmonary embolism, and atrial flutter on chronic anticoagulation with apixaban presented for new-onset general tonic-clonic seizures, witnessed by her family. The patient had been bed-bound from arthritis in a nursing home, but her mental status had been intact. The patient had recently been hospitalized over 3 weeks ago for COVID-19 pneumonia and was discharged home with home hospice one week before readmission.

**Figure 1:**
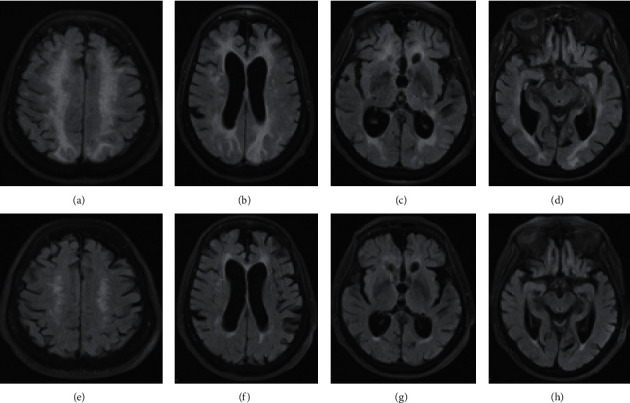
(a–d) Axial FLAIR images at multiple levels demonstrate cortical, subcortical, and periventricular signal hyperintensity and mild mass effect typical of PRES, predominantly involving the bilateral frontal, parietal, and occipital lobes on admission. Follow-up MRI (e–h) images four months after presentation showed resolution of the lesions.

According to her family, the patient's mental status had severely declined during her previous hospitalization with COVID-19. She developed staring spells after discharge that culminated into generalized tonic-clonic seizures on the day of the current admission. The episode lasted two seconds. Per her hospice nurse, blood pressures and blood glucose were well controlled at home. The home medications were thoroughly reviewed and found not to have any typical pharmacologic culprits. When she presented to the emergency department, she had two more witnessed seizures. Her blood pressure during this episode was 227/95 mm Hg, her heart rate was 98 beats/min, the temperature was 36.7°C, her respiratory rate was 16 breaths/min, and oxygen saturation was 93% while she breathed room air. Intravenous labetalol, lorazepam, and levetiracetam were administered. Her blood pressure decreased to 160/70 mm Hg and remained well controlled during her hospitalization. Head CT and basic laboratory work (Table 1) were unremarkable. Her physical exam was notable for postictal confusion. She was alert and only oriented to person. An electroencephalogram (EEG) detected no evidence of seizure or epileptiform discharges, but generalized slowing with an intermittent focal slowing in the bilateral temporal regions was noticed (Table 1). Brain MRI demonstrated subcortical and cortical FLAIR signal abnormality involving the left greater than right parieto-occipital lobes and the left temporal lobe, in a pattern most compatible with posterior reversible encephalopathy syndrome (PRES) (Figures 1(a)–1(d)). There was no acute intracranial hemorrhage or infarction.

The patient had no further seizures after being treated with levetiracetam. Her mental state gradually returned to normal. She became more eager to participate in physical therapy and be more independent. She set a goal to walk without her walker and to cook for her family and friends. Her family was pleasantly surprised by this improvement in her mentation. She had no further seizure activity in the hospital and was discharged back home with home care services. She is doing well six months after discharge, is seizure-free, and follows her scheduled appointments. Follow-up MRI four months later after presentation showed complete or near resolution of the lesions (Figures 1(e)–1(h)).

No written consent has been obtained from the patient as there is no patient identifiable data included in the case report.

## 3. Discussion

The pathogenesis of PRES is controversial and not always clearly defined [10–15]. We speculate that the COVID-19 virus-associated endothelial dysfunction progressed after the acute phase of the disease and likely contributed to PRES in our patient.

PRES has been described in COVID-19 patients in a few case-based observational studies set during the acute phase of COVID-19 hospitalization [1, 16, 17]. We believe that this is the first case describing the presentation of PRES after discharge from initial COVID-19 hospitalization, which may reflect a delayed neurological sequela of SARS-CoV-2 requiring readmission. Our patient did not receive any specific medications for COVID-19 infection but was treated in a clinical trial known as the RESCUE trial with chest single low-dose radiation therapy for severe pneumonia [18].

Our patient's blood pressure during her presentation was elevated only on one occasion after grand mal seizure but was well controlled throughout both hospitalizations and at home. A list of home medications was thoroughly reviewed and found not to have any typical pharmacologic culprits. Her recovery from severe COVID-19 infection-associated pneumonia, acute renal failure, and seizure from PRES was remarkable given her age and multiple comorbidities. Treatment of PRES depends on identifying any potential triggers and removing them. Our patient was treated with supportive care and investigational single low-dose chest radiation during her initial hospitalization [19]. We guess that PRES could be a potential complication of severe COVID-19 infection and in appropriate clinical settings, and PRES diagnosis may prompt the clinician to consider a diagnosis of COVID-19 in the acute or reconvalescent phase.

## Figures and Tables

**Table 1:** Clinical characteristics during initial hospitalization and readmission.

Variables	Initial admission	Readmission
Reason for admission	Fever	Seizure
Comorbidities	HTN, osteoarthritis, DM 2, VTE, CKD stage 2, atrial flutter, cataract, macular degeneration, right eye blindness, pressure ulcer stage II, mild dementia	HTN, osteoarthritis, DM 2, VTE, CKD stage 2, atrial flutter, cataract, macular degeneration, right eye blindness, pressure ulcer stage II, mild dementia
COVID-19 PCR	Positive At discharge: negative	Not tested
Symptoms of COVID-19	Fever and cough, acute, respiratory failure, altered mental status	NA
Hospital problem list	Acute respiratory failure, viral pneumonia, acute diffuse body pain, DM 2, HTN, acute renal failure, delirium, cystitis, hypokalemia	New-onset seizure, acute metabolic encephalopathy, cystitis, hypomagnesemia, hypokalemia
Risk factors for PRES	N/A	BP fluctuations, recent COVID-19 infection
The onset of PRES from diagnosing COVID-19	NA	31 days
LOS, total	22 days	6 days
LOS, ICU	2 days	0
LOS, floor	20 days	6 days
MAP, range	81–120	91–113
SBP, range	124–192	127–227
Medications	Acetaminophen, apixaban, ceftriaxone, insulin, labetalol, ondansetron, magnesium sulfate, potassium chloride, hydralazine, gabapentin, tramadol	Acetaminophen, apixaban, labetalol, ceftriaxone, insulin, levetiracetam, tramadol (stopped at admission)

Specific treatment for COVID-19	Enrolled in RESCUE trial: received single low-dose chest radiation	
